# Application of preclinical absorption, distribution, metabolism, elimination in vitro techniques for the characterization and compound library optimization of novel antibiotic gallium salophen

**DOI:** 10.1016/j.dmd.2025.100080

**Published:** 2025-04-16

**Authors:** Samuel A. Krug, Aziza Frank, Lucia Hwang, Madison Worth, Kieran Johnson, Christine Rojas, Ludovic Muller, Sarah L.J. Michel, Angela Wilks, Fengtian Xue, Maureen A. Kane

**Affiliations:** Department of Pharmaceutical Sciences, School of Pharmacy, University of Maryland, Baltimore, Maryland

**Keywords:** Absorption, distribution, metabolism, elimination, Antibiotic, ICP-MS, LC-MS/MS, Metallotherapeutic, *Pseudomonas aeruginosa*

## Abstract

Multidrug-resistant pathogens are an emerging threat to public health. Metal-based drugs have shown antimicrobial properties. As a result, metallotherapeutics have an untapped potential to combat antibiotic-resistant infections. Characterization of additional physiochemical attributes is needed to progress metallodrugs in the clinical pipeline. In order to fully characterize a modest library of compounds based on novel therapeutic gallium salophen (GaSal), target binding affinity (dissociation constant, K_d_), lipophilicity (octanol-water partition coefficient, logP), protein binding (fraction of protein bound compound, K_b_), Caco-2 permeability, microsomal stability, and blood/plasma partitioning (blood-plasma partition coefficient, K_RBC__/PL_) experiments were performed to help inform lead optimization. Analogs with 2 identical solubilizing groups had a lower theoretical LogP than analogs with only one solubilizing group; however, the experimental data showed the inverse to be true. All of the analogs tested were highly plasma protein bound (>98%). Caco-2 permeability showed that the apparent permeability from apical to basolateral had limited permeability. Apparent permeability calculated from the basolateral to apical side, however, resulted in a high efflux ratio for all compounds. The addition of inhibition cocktails for P-glycoprotein and organic cation transporter 1 did not vastly impact the efflux ratio, indicating that further investigation is needed to determine the transporter involved in drug distribution. Microsomal stability results indicated that GaSal analogs do not undergo cytochrome P450 metabolism, likely are metabolized by enzymes found in the S9 liver fraction (S9), and can potentially be cytochrome P450 inhibitors. This study also provides insight into optimizing liquid chromatography and mass spectrometry parameters because metallodrugs show unique ionization and physiochemical properties. Finally, to our knowledge, this article is the first to detail Ga^3+^ blood/plasma partitioning because this is not a common metal found in diet or environmental exposure. During partitioning experiments, analogs with polar acidic functional groups portioned heavily into the red blood cells compared to other analogs. Herein, we determine in vitro physiochemical properties in order to characterize absorption, distribution, metabolism, elimination parameters useful for subsequent generations of GaSal analogs as metallotherapeutics.

**Significance statement:**

Few studies detail drug metabolism and pharmacokinetic (DMPK) library screening for metal-based therapeutics, and there is a large literature gap in metallodrug preclinical development. Establishing the relationship between inductively coupled plasma mass spectrometry and liquid chromatography tandem mass spectrometry analysis is critical during preclinical development to ensure in vitro pharmacokinetic parameters are accurately reported. The information from this work is important for optimizing gallium salophen analogs as potential metallotherapeutics against multidrug-resistant pathogens.

## 1 Introduction

Deaths related to antimicrobial resistance are expected to surpass cancer by the year 2050 (Dadgostar, 2019). Despite this emerging threat, very few novel antibiotics have come to market in the past decade (Akram et al, 2023). This is particularly concerning for pathogens, such as *Pseudomonas aeruginosa* (Pa), that are highly adaptable to their environment, remain a common nosocomial infection, and are persistent in immunocompromised patients (De Oliveira et al, 2020; Reynolds and Kollef, 2021; Kunz Coyne et al, 2022). In order to combat multidrug-resistant (MDR) pathogens, combination therapy such as piperacillin/tazobactam is used to address *β*-lactamase upregulation due to environmental stress (CLSI Rationale Document MR15, 2024). Recently, Clinical Laboratory Improvement Amendments have addressed an increase in clinical isolates resistant to combination therapy, and MDR pathogens demonstrate unique regional trends (Ullah et al, 2019).

**Fig. 1:**
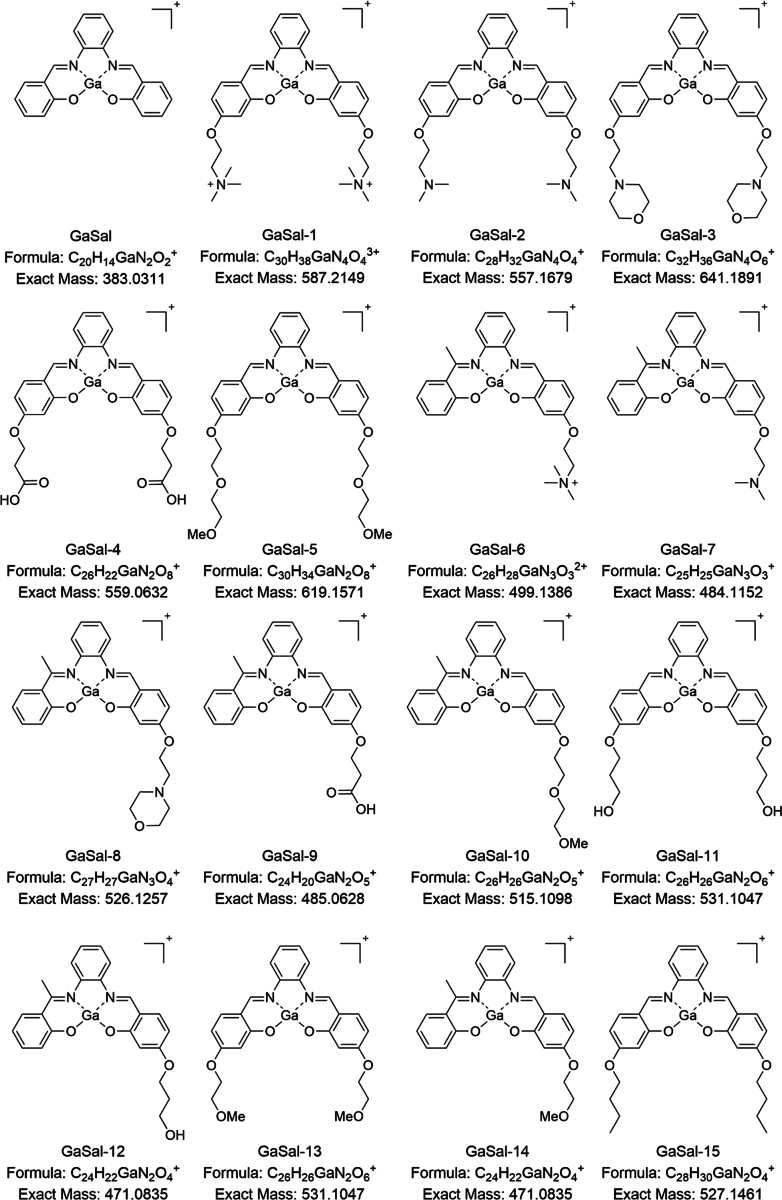
Structures, formula, and predicted exact mass of GaSal library compounds tested.

*Pseudomonas aeruginosa* requires iron for virulence which can be acquired by siderophores that bind free iron or heme (Behzadi et al, 2021). Targeting *P*. *aeruginosa* iron uptake systems has been attempted; however, this treatment regime results in the generation of persister cells and increased production of siderophores (Choi et al, 2020). Our laboratories have demonstrated that a novel antibiotic, gallium salophen (GaSal) is able to interfere with heme sensing through the *P*. *aeruginosa* heme assimilation system (HaS) by binding with HasAp, generating a lead structure for further optimization (Centola et al, 2020). The subsequent generation of compounds included structural moieties to enhance solubility; library compounds described in this article are shown in Fig. 1. Synthesis and characterization of these compounds are described in detail previously and in ongoing studies (Centola et al, 2020; Frank et al, 2022). Although this drug candidate has shown promise for in vitro testing in *P*. *aeruginosa* reference strain PAO1, further in vitro characterization is needed to understand the physiochemical properties of GaSal in the host.

Since the Food and Drug Administration approval of cisplatin, medicinal inorganic chemistry has been applied for antimicrobial, antiviral, and anti-inflammatory metallodrug development (Frei et al, 2020). Many of these drug programs do not successfully translate to the clinic because of ambiguity in regulatory guidance and challenges with preclinical screening (Anthony et al, 2020). Metals may present unique challenges for common preclinical screening and analytical methods. For example, during high throughput target screening, which can rely on fluorescence to indicate binding with the target, metals can present potential interference with wavelength absorption and emission. Screening to assess this potential effect is a necessary step in preclinical assay development for metallotherapeutics. Similar screening needs to be performed with colorimetric agents used to assess cell proliferation and viability (Cai et al, 2019). Metals are abundant in cell culture media, so it is necessary to assess whether transmetalation is occurring, thus disrupting the intact drug molecule (Haase et al, 2015; Levina et al, 2017). Furthermore, it has been demonstrated that DMSO can interfere with platinum-based drugs, so it is also necessary to consider solvent composition (Hall et al, 2014). Methods of analysis for pharmacokinetic (PK) determination of metallodrugs may also present class-distinct challenges and need to be thoroughly explored. Inductively coupled plasma mass spectrometry (ICP-MS) analysis is frequently used; however, there has been literature that demonstrates the PK profile for platinum is different than the PK profile for cisplatin, carboplatin, and oxaliplatin (Qin et al, 2019). This underscores a need to establish the relationship between quantitative values obtained by ICP-MS as a surrogate of the drug molecule and liquid chromatography tandem mass spectrometry (LC-MS/MS) quantitative values of the drug molecule itself during initial drug characterization. Together, these factors create distinct hurdles in metallodrug development.

Preclinical in vitro studies help to optimize drug candidates and inform on drug properties that need to be determined early in the clinical pipeline. These properties include lipophilicity, protein binding, permeability, stability, and whole blood partitioning. Lipophilicity, or logP, can influence the distribution of drugs into different compartments of the body. When a drug is highly protein bound, it reduces the availability to reach the therapeutic target and can affect the dose needed to achieve a therapeutic response. Caco-2 permeability is used to assess the apparent permeability (P_app_) across the intestinal barrier (Hubatsch et al, 2007). This can provide insight into the potential oral bioavailability of the compound as well as any transporter-mediated interactions that need to be further characterized before advancing the drug program. Microsomal stability can help to further define the biotransformation of the compounds and indicate how quickly the drug is cleared from the system (Li et al, 2006). Finally, blood/plasma (PL) distribution is understudied and underreported in the literature; however, this information is underused and can be used to provide parameters for physiologically based PK modeling during preliminary toxicology and clinical studies (Hinderling, 1997; Poulin et al, 2024). Together, these assays provide an ensemble of information used to further optimize lead compounds.

Within this article, we have applied lipophilicity, protein binding, Caco-2 permeability, microsomal stability, and whole blood partitioning to better understand the distribution and metabolism of drugs in the GaSal program. This work will help to progress the knowledge of metallodrug development and highlight quality control considerations for the clinical development of metal-containing therapeutics.

## 2 Materials and methods

### 2.1 Compound synthesis

The following library compounds were synthesized in the library of Fengtian Xue at the University of Maryland, Baltimore: GaSal, GaSal1, GaSal2, GaSal3, GaSal4, GaSal5, GaSal6, GaSal7, GaSal8, GaSal9, GaSal10, GaSal11, GaSal12, GaSal13, GaSal14, and GaSal15.

### 2.2 Expression and purification of wild-type apo-HasAp

Wild-type, full-length HasAp was prepared from freshly transformed *Escherichia coli* BL21(DE3) competent cells as previously described in 4 L of M9 media. Cell pellets were resuspended and lysed with an LM-20 microfluidizer at 18,000 psi. Lysate was clarified by centrifugation and applied to a Q-Sepharose column. HasAp was eluted over a gradient from 20 to 600 mM NaCl in 20 mM Tris-HCl (pH 7.5), and desired fractions determined by SDS-PAGE were pooled. Apo-HasAp was further purified on a Butyl Sepharose Fast Flow column equilibrated with 50 mM sodium phosphate buffer with 0.7 M ammonium sulfate (pH 7.0). Holo-HasAp and other proteins eluted within 2–3 bed volumes of 50 mM sodium phosphate containing 0.5 M ammonium sulfate. Apo-HasAp was then eluted with a linear gradient of sodium phosphate buffer (50–20 mM) containing ammonium sulfate (0.5–0 M). Fractions were analyzed again by SDS-PAGE, and the apo-HasAp was pooled, concentrated (Amicon stirred cell with Ultracel 10 kDa filter), and exchanged into 20 mM sodium phosphate buffer.

### 2.3 K_d_ determination for GaSal analogs using fluorescence quenching

Binding affinities were determined using a BioTek Synergy H1 microplate reader in a black 96-well plate. Compounds were solvated in H_2_O in serial dilutions (100–0.39 *μ*M). One micromolar solution of apo-HasAp 20 mM sodium phosphate buffer (pH 7.4, 25 °C) was titrated into the ligand of interest. Emission spectra were recorded (300–500 nm) after excitation at 280 nm. The decrease in maximum emission was plotted against the ligand concentration (accounting for dilution) and fit to one-site binding using GraphPad Prism v10.

### 2.4 ICP-MS analysis

Biological samples of PL, red blood cells (RBCs), and whole blood were prepared for ICP-MS analysis by initial digestion in concentrated (67%) nitric acid up to a total volume of 250 *μ*L. The mixtures were digested at 80 °C for 4 hours and further diluted to a concentration of 6% nitric acid. Gallium (Ga^71^) in whole blood, PL, and RBC samples were quantified using an Agilent 7700x ICP-MS instrument (Agilent Technologies). Metal levels were detected using an Octopole Reaction System in He mode. Other ICP-MS parameters include an radio frequency power of 1500 W, on octupole radio frequency of 190 V, an octapole bias of –18 V, a helium gas flow of 4.3 mL/min, and an argon flow of 0.99 L/min. Samples were loaded through a 7700X peristaltic (with a speed of 0.1 rps) and a MicroMist nebulizer. A standard calibration curve was used to derive metal concentrations. The calibration standards from 0–250 ppb were prepared in 6% nitric acid using atomic absorption standards (Sigma). Data analysis and extraction were performed using Agilent’s MassHunter software version 5.1, specifically version D.01.01.

### 2.5 LC-MS/MS analysis

All LC-MS/MS grade solvents were purchased from ThermoFisher. Ammonium bicarbonate was purchased from Sigma Aldrich. Analysis was performed on a Waters I-Class ultra-performance liquid chromatography system connected with a Waters Xevo TQ-XS mass spectrometer. Ten millimolar ammonium bicarbonate, pH 9.4 was used for mobile phase A, and 100% methanol was used for mobile phase B. The column flow rate was set at 0.400 mL/min. Chromatographic separation was performed with a BEH C18 (2.1 × 50 mm, 1.7 *μ*m) column (Waters). The initial gradient was set to start at 40% B for 0.5 minutes, ramp to 95% B over 1.5 minutes, and held at 95% B for 1 minute, and then return to 40% B over 0.5 minutes and held for 0.5 minutes. The total run time was 4 minutes. The column temperature was set to 60 °C, and the autosampler temperature was set to 15 °C. Washing parameters were 800 *μ*L of weak needle wash (acetonitrile [ACN]:Water/50:50) and 1000 *μ*L of strong needle wash (methanol:ACN:2-propanol:formic acid/30:30:40:0.5) after every injection. Transitions used to monitor all drugs in the study are shown in Supplemental Table 1.

### 2.6 Desorption electrospray ionization analysis

Desorption electrospray ionization (DESI) mass spectrometry (MS) experiments were carried out using a high definition mass spectrometry (HDMS) SYNAPT G2-Si mass spectrometer with Tri-Wave technology (Waters Corp), Cyclic IMS (Waters Corp), and a high precision XY stage moving the sample surface under the spray for a pixel size 60 *μ*m. A mixture of methanol and water (95:5 v/v, 0.1% formic acid) as a spray solvent was used. All MS data were acquired in positive ion mode and in the resolution MS mode with an m/z range from 200 to 1200.

### 2.7 LogP determination

LogP was experimentally determined using previously published reverse-phase high-performance liquid chromatography method, and this workflow was adapted using instrumentation described in the previous LC-MS/MS analysis section (Coutinho et al, 2023). Briefly, analogs were eluted using isocratic flow with an increasing percentage of organic solvent from 55% to 90% in increments of 5%. The change in the log of the retention factor, log k, was plotted against the fraction of organic solvent. This allowed for extrapolation to the y-intercept, which is when the fraction of organic in the system is zero, or log k_w_. The same workflow was applied to known compounds with a published logP value to establish a relationship between logP and log k_w._ This ratio was used to calculate the experimental logP from the log k_w_ of the test compounds. The in silico prediction of logP values was obtained using an online calculator by Molinspiration Cheminformatics (molinspiration.com).

### 2.8 PL protein binding

The fraction of test compound bound to PL proteins was determined using a rapid equilibrium dialysis device with 8000 molecular weight cut-off (ThermoFisher). Inserts were soaked in deionized water for 10 minutes, and then, the water was refreshed and allowed to sit for another 10 minutes before starting the experiment. All compounds were tested at a concentration of 5 *μ*M. Two hundred microliters of the test compound in human PL were placed on the donor side, whereas 350 *μ*L of PBS, pH 7.4 was added to the receiver chamber. All samples were run in duplicate. The plate was sealed with an adhesive strip and shaken at 250 rpm at 37 °C for 4 hours using an Accu-Therm plate shaker (LabNet). An aliquot of 50 *μ*L was taken from the donor and receiver sides, and the samples were matrix matched by adding 50 *μ*L blank PL to the receiver samples or 50 *μ*L of PBS, pH 7.4 to the donor samples. Samples were extracted using 300 *μ*L ACN for protein precipitation. Samples were vortexed for 1 minute at high speed and then centrifuged at 4000 rpm at 4 °C for 10 minutes. The supernatant (200 *μ*L) was transferred to a new 96-well plate using a Hamilton MicroPREP system, and 100 *μ*L of LC-MS/MS grade water was added to each sample before analysis. The fraction of the unbound compound was calculated by dividing the internal standard-normalized peak area of the receiver chamber by the IS-normalized peak area of the donor chamber. Methotrexate, verapamil, and diazepam were used as control compounds in this experiment with expected protein binding of 50%, 90%, and 99%, respectively (Zhang et al, 2012).

### 2.9 Caco-2 permeability screening

Culturing and screening are briefly described here; cells were cultured as previously described for Caco-2 permeability assessment (Hubatsch et al, 2007). Caco-2 cells were received from American Type Culture Collection and certified mycoplasma free. Cells were seeded on transwell plates (CoStar, growth area 0.33 cm^2^/well, polycarbonate membrane, 0.33 *μ*m pore size) between passage 20 and 27 and then analyzed between 21 and 25 days after wells were seeded to ensure enterocyte-like differentiation. Before assessing permeability, cells were washed once with Hank’s Balanced Salt Solution buffer, pH 7.4, and then incubated at 37 °C for 30 minutes. Transepithelial electrical resistance (TEER) was measured after equilibration before the experiment using an epithelial volt-ohmmeter (EVOM, World Precision Instruments), and cells were used if TEER > 250 Ω × cm^2^. Cells were dosed to measure permeability from basolateral and apical sides at a concentration of 20 *μ*M of test compound and 50 *μ*M of Lucifer yellow. Lucifer yellow was used as a “zero permeability” marker to monitor the integrity of cell barrier. Samples were taken at 0 and 60 minutes from the donor side, and samples were taken at 30 and 60 minutes from the receiver side; media was replenished after the 30-minute sample was taken from both chambers. A calibration curve was prepared by serial dilution using the dosing solution in a 1:5 dilution. At the end of incubation, TEER was recorded, and then, the plate was read for Lucifer yellow permeability as previously described (Corning Life Sciences, 2012). Samples were prepared for LC-MS/MS analysis by taking 100 *μ*L of sample, adding 1 *μ*M internal standard (GaSal-4 or GaSal-5), and crashing with 400 *μ*L of methanol. Samples were centrifuged at 3000 rpm at 4 °C for 30 minutes, 200 *μ*L of supernatant was transferred to a new plate, and 100 *μ*L of water was added to each sample. Samples were vortexed and analyzed by LC-MS/MS as described earlier. Samples were then compared to known low (methotrexate), moderate (loperamide), and high permeability (citalopram) control drugs. To preliminarily evaluate organic cation transporter 1 (OCT1) or P-glycoprotein (P-gp)-dependent transport, transport studies were conducted with and without a 1 *μ*M “inhibitor cocktail” alongside GaSal. The P-gp inhibitor cocktail consisted of cannabidiol and itraconazole (both from Cerilliant), whereas the OCT1 inhibitor cocktail comprised verapamil and cisplatin (both from Sigma). The P_app_ for basolateral to apical, P_app_ apical to basolateral, and efflux ratio were reported for each compound. In order to calculate P_app_, the concentration in the sampled time point was determined from the calibration curve and then converted to *μ*g based on the volume of the chamber (0.2 mL for apical, 1.0 mL for basolateral). Mass balance was assessed by comparing the amount of drug in the dosing chamber at the start and end of the incubation period with the amount calculated from receiver sampling and considered acceptable if the mass balance was between 80% and 120%. The calculation of P_app_ was determined using eq. 1 as follows:

(1) $$P_{app}=\frac{dQ}{dt}*\frac{1}{\left(A\;x\;C_{0}\right)}$$

Where dQ/dt is the rate of drug permeation (slope of the amount of drug vs time), A = 0.33 *μ*m for our transwell system, and C_0_ is the initial concentration. Data are presented as the average ± SEM for each compound analyzed as *n* = 3 on 2 different days with 2 different cell line passages, for *n* = 6 total. GaSal is *n* = 3 on 3 different cell line passages since this was used as a test compound for the inhibition cocktail experiments.

#### 2.9.1 Microsomal clearance assay

Library compounds were tested in triplicate at a concentration of 2 *μ*M and incubated in 0.1 M potassium phosphate buffer containing 12 mM MgCl_2_ and 8 mM NADPH at pH 7.4 with either 0.5 mg/mL of Sprague–Dawley liver microsomes (Invitrogen). Samples were incubated for 0, 3, 7, 12, 20, and 30 minutes. At the designated time point, the incubation reaction was stopped by adding 100 *μ*L of ACN containing 1 *μ*M of either GaSal-4 or GaSal-5 as the internal standards. Samples were then centrifuged at 4000 rpm at 4 °C for 10 minutes, and 100 *μ*L of supernatant was taken for LC-MS/MS analysis. 7-Ethoxycoumarin (Sigma) was used as a positive control in this experiment. Intrinsic clearance (CL_int_) was calculated using eq. 2:

(2) $${CL}_{\mathrm{int}}=\frac{0.693}{In\;Vitro\;T_{\frac{1}{2}}}*\frac{Incubation\;Volume}{mg\;of\;microsomes}*\frac{45\;mg\;microsomes}{gram\;of\;liver}*\frac{45\;grams\;liver}{kg\;of\;body\;weight}$$

The average CL_int_ ± SEM was determined for each compound for 2 replicates per time point.

#### 2.9.2 S9 clearance assay

Select library compounds were tested in triplicate at a concentration of 2 *μ*M and incubated in 0.1 M potassium phosphate buffer containing 12 mM MgCl_2_ and 8 mM NADPH at pH 7.4 with 2.5 mg/mL of Sprague–Dawley S9 (Invitrogen). Incubations contained a final concentration of 2.5 mM UDP glucuronic acid, 0.10 mM adenosine 3’-phosphate 5’-phosphosulfate, 0.5 mM acetyl CoA, and 50 μg/mg protein alamethicin. All co-factors were from Sigma. Samples were incubated for 0, 3, 7, 12, 20, and 30 minutes. At the designated time point, the incubation reaction was stopped by adding 100 *μ*L of ACN containing 1 *μ*M of either GaSal-4 or GaSal-5 as the internal standard. Samples were then centrifuged at 4000 rpm at 4 °C for 10 minutes. A hundred microliter of supernatant was taken for LC-MS/MS analysis. 7-Ethoxycoumarin (Sigma) was used as a positive control in this experiment. Clearance was calculated according to eq. 3:

(3) $${CL}_{\mathrm{int}}=\frac{0.693}{In\;Vitro\;T_{1/2}}*\frac{Incubation\;Volume}{mg\;of\;S9}*\frac{165\;mg\;S9}{gram\;of\;liver}*\frac{45\;grams\;liver}{kg\;of\;body\;weight}$$

The average CL_int_ ± SEM was determined for each compound for 3 replicates per time point.

#### 2.9.3 RBC/PL partitioning equilibrium

Whole blood (either potassium EDTA [K_2_EDTA] or sodium heparin) was preincubated at 37 °C for 30 minutes before spiking with the analyte. GaSal-5 was made at a stock concentration of 100 *μ*g/mL in water and then spiked at a high concentration (1 *μ*g/mL) and low concentration (100 ng/mL) to determine whether equilibrium was concentration dependent. Samples were then aliquoted and stored in a water bath at 37 °C for 1, 2, 3, 4, and 8 hours or harvested immediately. PL was harvested from samples by centrifuging at 3000 rpm at 4 °C for 10 minutes. PL was transferred to a new tube and considered the PL fraction, whereas the rest of the matrix was considered the RBC fraction. Samples were then either analyzed by LC-MS/MS or ICP-MS. For library screening, samples were prepared for GaSal analogs at high and low concentrations in both K_2_EDTA and sodium heparin-treated whole blood; however, they were only incubated and harvested after 2 hours because this was the determined equilibration time from the previous experiment. For selectivity lot analysis, whole blood from 3 male and 3 female donors were incubated for 2 hours and then analyzed with LC-MS/MS and ICP-MS at a single concentration of 1 *μ*g/mL. Data are presented as average ± SEM, *n* = 3 per sample, per concentration. Significance was determined by using a paired *t* test for ICP-MS and LC-MS/MS data (the same sample, different method) or a 2-tailed Student’s *t* test for different anticoagulants.

## 3 Results

### 3.1 LC-MS/MS optimization

LC-MS/MS analysis poses unique challenges for metallodrug development. With the original GaSal parent drug, we noticed high variation when injecting from the same vial with a reconstitution solvent of 90:10 (water:ACN); however, increasing the ratio of organic solvent in the reconstitution solution resolved this issue. The increase of organic solvent in the reconstitution then posed issues for the retention of the analyte on the column. We found that using methanol as the organic solvent increases the retention of the analyte on the column as well as using a slightly basic mobile phase (pH ∼9.4). These changes resulted in a retention factor (k’) >1 which demonstrates acceptable retention of the metallodrug on the analytical column. The GaSal library carries a positive charge due to the metal core, so unlike typical molecules where the [M + H]^+^ or [M – H]^–^ peak is analyzed, the M^+^ peak is analyzed for MS1. This is an important distinction because if an analyst is using automated MS/MS fragment generation, they are likely to optimize based on the C-13 peak. The typical adducts found for electrospray ionization + analysis mode are H^+^, Na^+^, K^+^, and NH_4_^+^; however, with metallodrugs, we see characteristic peaks of additional Ga^3+^ and ligands adducts. This is consistent with other reported metallodrug spectra (Huang et al, 1997). When comparing electrospray ionization and DESI ionization, the same metal adducts did not exist in the DESI spectra (Supplemental Fig. 1). Finally, it is possible to induce in-source dissociation if declustering potential or cone voltage is too high (Supplemental Fig. 2). We used a GaSal analog as the internal standard because it will display similar ionization, solubility, and physiochemical properties. Tertiary amine analogs, GaSal-2 and GaSal-7, showed a notable trend important for assays such as the Caco-2 permeability screening. Whereas these compounds can be detected by LC-MS/MS analysis in a neat solution, in a biorelevant media, these compounds are not detected. For these tertiary amine analogs, detection of gallium by ICP-MS analysis for partitioning experiments showed a similar distribution to other analogs.

#### 3.1.1 K_d_, LogP, and protein binding assessment

Table 1 summarizes the data collected using techniques to calculate K_d_, LogP, and K_b_ for the library. The K_d_ values of all compounds to the target protein HasA ranged from 0.33 to 5.0 *μ*M, showing that the addition of solubilizing moieties to the salophen core had little effect on the binding. Interestingly, the experimental LogP showed the opposite trend compared to the predicted LogP model, whereas the in silico approach predicted that the analogs with 1 solubilizing moiety would be more lipophilic than analogs with 2 solubilizing groups. GaSal-3 and GaSal-8, which have the morpholine solubilizing group, were the only 2 analogs that were consistent with in silico prediction. Example chromatograms demonstrating retention time shift for GaSal-3 are shown in Supplemental Fig. 3. PL protein binding showed that all analogs are highly PL protein bound with K_b_ > 0.98.

**Table 1:** GalSal analog properties Summary table for GaSal library compounds with experimentally determined target binding via dissociation constant (K_d_), lipophilicity via octanol-water partition coefficient (LogP), and plasma protein binding via fraction of protein bound compound (K_b_)

Compound ID	Scaffold	HasA K_d_ (*μ*M)	LogP In Silico	LogP Experimental	K_b_
GaSal (Parent)	Two-armed	0.80 ± 0.10	2.93	2.58	>0.99
GaSal-1	Two-armed	2.5 ± 1.2	–6.01	n.d.^a^	n.d.^a^
GaSal-2	Two-armed	1.2 ± 0.3	3.31	3.70	n.d.^b^
GaSal-3	Two-armed	1.5 ± 0.3	3.27	3.87	>0.98
GaSal-4	Two-armed	1.1 ± 0.1	2.21	3.33	>0.99
GaSal-5	Two-armed	2.5 ± 1.2	2.54	3.95	>0.99
GaSal-6	One-armed	4.0 ± 2.8	0.03	n.d.^a^	n.d.^a^
GaSal-7	One-armed	5.0 ± 0.9	3.99	3.27	n.d.^b^
GaSal-8	One-armed	5.2 ± 0.4	4.01	4.11	>0.99
GaSal-9	One-armed	0.33 ± 0.04	3.44	3.03	>0.99
GaSal-10	One-armed	3.4 ± 0.3	3.61	n.d.^a^	n.d.^a^
GaSal-11	Two-armed	0.57 ± 0.03	2.15	4.04	>0.99
GaSal-12	One-armed	0.52 ± 0.01	3.42	3.19	>0.99
GaSal-13	Two-armed	0.96 ± 0.14	2.89	3.65	>0.99
GaSal-14	One-armed	2.84 ± 0.12	3.79	3.31	>0.99
GaSal-15	Two-armed	1.98 ± 0.17	6.20	5.99	>0.99

n.d.^a^, not determined due to compound availability; n.d.^b^, not determined due to poor LC-MS/MS signal.

#### 3.1.2 Caco-2 permeability

Four drugs that are well characterized for Caco-2 permeability were used as controls with this experiment and were in good agreement with previously published results (Sevin et al, 2013; Kamiya et al, 2020). Permeability from lowest to highest for our known compounds is methotrexate, minoxidil, loperamide, and citalopram; calculated P_app_ is included in Supplemental Table 2. Based on the P_app_ values obtained from the control drugs, our library compounds were ranked as follows: not permeable, P_app_ test compound < P_app_ minoxidil; low permeability, P_app_ minoxidil < P_app_ test compound < P_app_ loperamide; moderate permeability, P_app_ loperamide < P_app_ test compound < P_app_ citalopram; and high permeability, P_app_ citalopram < P_app_ test compound. The results of these experiments are shown in Table 2. Notably, all of the analogs apart from the parent compound are not permeable from the apical to the basolateral side; however, there is a wide range of permeability from the basolateral to the apical side. All drugs were screened at 15-, 30-, and 60-minute time points; however, 15 minutes was not a long enough incubation for there to be a quantifiable drug signal for a majority of the compounds. In order to be consistent with reported time points, 30 and 60 minutes were used for P_app_ determination. For drugs listed with a P_app_ < 0.01, there was not a quantifiable signal for the 30-minute receiver sample. Two analogs listed as “Undetected” showed no signal in both the donor and receiver samples. Because the efflux ratio is >2 for a majority of the library, Caco-2 permeability was repeated for GaSal in the presence of a P-gp inhibitor cocktail and an OCT1 inhibitor cocktail. The P-gp inhibitor cocktail showed little impact on the transport of GaSal; however, the OCT1 inhibitor cocktail showed an increase in the apical to basolateral permeability of the drug (Supplemental Table 3). P-gp was chosen because this is a major efflux transport with high abundance in Caco-2 cells. OCT1 was selected because there is a positive charge on the coordinated metal. Prior to conducting these experiments, Lucifer yellow emission was evaluated in the presence of GaSal analogs and showed no impact; representative data are shown in Supplemental Fig. 4.

**Table 2:** Caco-2 permeability Data summary of Caco-2 permeability for GaSal library. Low, moderate, and high permeability were classified based on P_app_ values of low, moderate, and high permeable commercially available drugs used as a control with this cell line (data shown in Supplemental Table 2). Units of apparent permeability are ×10^-6^ cm/s. Data are presented as the average ± SEM for each compound analyzed as *n* = 3 on 2 different days with 2 different cell line passages

Analyte	P_app_ apical to basolateral	Permeability ranking	P_app_ basolateral to apical	Efflux ratio B to A/A to B
GaSal∗	1.20 ± 0.01	Low	13.6 ± 1.78	11.3
GaSal2	Undetected	N/A	Undetected	N/A
GaSal3	0.04 ± 0.01	Not permeable	1.16 ± 0.09	29
GaSal4	<0.01	Not permeable	1.44 ± 0.19	N/A
GaSal5	0.03 ± 0.01	Not permeable	3.2 ± 0.2	107
GaSal7	Undetected	N/A	Undetected	N/A
GaSal8	<0.01	Not permeable	5.75 ± 0.37	>5.75
GaSal9	<0.01	Not permeable	22.48 ± 5.63	>22.5
GaSal11	<0.01	Not permeable	0.72 ± 0.12	N/A
GaSal12	0.05 ± 0.01	Not permeable	0.17 ± 0.06	3.4
GaSal13	0.61 ± 0.01	Not permeable	0.91 ± 0.1	1.49
GaSal14	<0.01	Not permeable	1.19 ± 0.4	N/A
GaSal15	0.79 ± 0.06	Not permeable	7.1 ± 1.13	9.02

∗ *n* = 3 on 3 different days with 3 cell line passages.

#### 3.1.3 Microsomal and S9 clearance assays

**Fig. 2:**
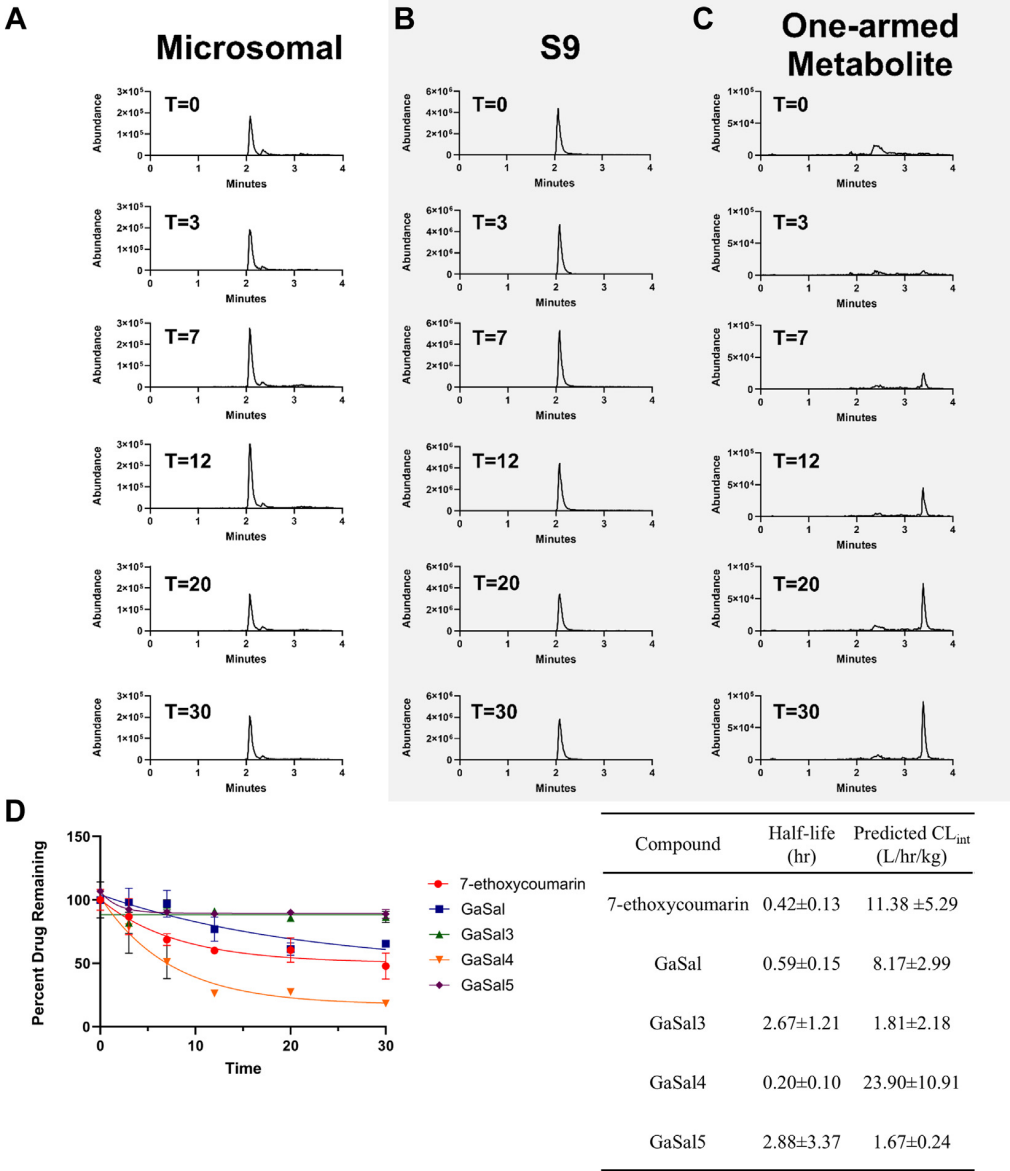
Microsomal and S9 stability. Representative chromatograms for microsomal and S9 fraction stability data. The left column (Panel A) shows GaSal-3 incubated in rat liver microsomes over a 30-mintue period with <10% decrease in peak area over time. The middle column (Panel B) is GaSal-3 incubated in S9 rat liver fraction with a decrease in peak intensity from 6 × 10^6^ counts to 4 × 10^6^ counts. The right column (Panel C) shows the appearance of a metabolite with the same mass transition as GaSal-8 during GaSal-3 incubation in S9 liver fraction. Bottom left graph (Panel D) shows the elimination slope calculated from incubation of GaSal analogs in S9 rat liver fraction and the resulting table (bottom right) shows the calculated predicted CL_int_ from the S9 rat liver fraction elimination data. Data are presented as average ± SEM with *n* = 3 per compound.

Microsomal clearance showed that the concentration of GaSal analogs remained unchanged over 30 minutes, whereas 7-ethoxycoumarin was metabolized by the microsomes. The parent drug is slightly metabolized, with an experimental CL_int_ of 1.28–3.78 L/hr/kg. The rest of the analogs analyzed showed no change from T = 0 and are reported as <0.85 L/hr/kg. In order to characterize if GaSal potentially undergoes non-cytochrome P450 (CYP)-related metabolic activity, GaSal, GaSal-3, GaSal-4, and GaSal-5 were incubated in S9s. CL_int_ for S9 metabolism ranged from 1.67–23.9 L/hr/kg. Example chromatograms are provided in Fig. 2 for the incubation of GaSal-3 in microsomes (2A) and S9 fraction (2B). Figure 2C shows the formation of a metabolite in the S9 fraction over the incubation period. In this example, the metabolite of GaSal-3 shares the same mass transition as GaSal-8; however, the retention time is different. Counterintuitively, the elution time for the metabolite is later than the parent compound, indicating an increase in lipophilicity of the metabolite, whereas metabolic processes typically result in a more polar metabolite to aid in elimination. 7-Ethoxycoumarin was used as the positive control and agreed with previously reported CL_int_ for microsomes; however, 7-ethoxycoumarin, when incubated in the presence of all of the GaSal analogs, was not metabolized, indicating possible CYP inhibition.

#### 3.1.4 Whole blood partitioning

Initial partition equilibrium time experiments were performed in both K_2_EDTA for both gallium nitrate and GaSal-5 as a representative analog. Gallium nitrate showed >5% hemolysis at longer time points. Gallium nitrate equilibrium data measured by ICP-MS demonstrated that equilibration time is not concentration dependent and K_RBC/PL_ = 0.115 ± 0.010 and 0.114 ± 0.026 for low-concentration quality control sample (LQC) and high-concentration quality control sample (HQC) levels, respectively. A comparison of previously reported b/p values for gallium from this study and other metals from the literature is shown in Supplemental Table 4. Two hours was determined to be the optimal equilibrium time based on the level of hemolysis of the sample, the precision between replicates, and minimal change between the 2- and 3-hour time points.

**Fig. 3:**
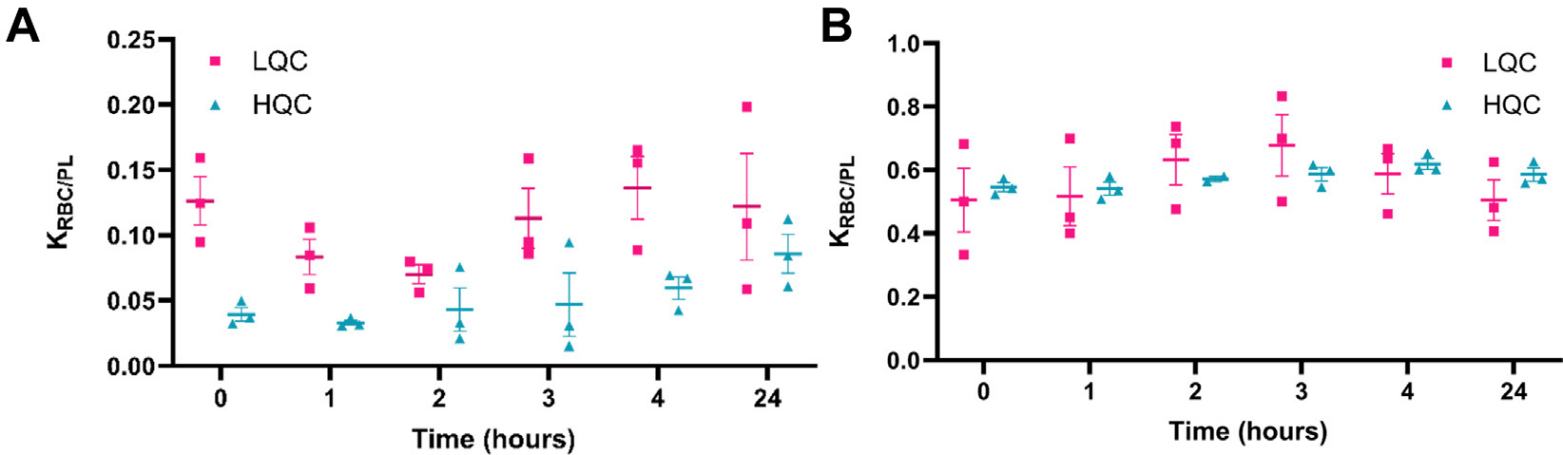
RBC/PL partitioning equilibrium. Data from whole blood partitioning experiments. Comparison of equilibrium time of GaSal5 in K_2_EDTA treated whole blood by LC-MS/MS (A) and ICP-MS (B) shows variability in LC-MS/MS samples measuring the intact drug compared to monitoring gallium only. Data are presented as average ± SEM, *n* = 3 per sample, per concentration.

When comparing ICP-MS with LC-MS/MS equilibrium data for GaSal-5, the K_RBC/PL_ at 2 hours was 0.070 ± 0.007 (LQC) and 0.043 ± 0.017 (HQC) for the LC-MS/MS samples, whereas the same samples analyzed by ICP-MS gave a K_RBC/PL_ of 0.632 ± 0.080 (LQC) and 0.572 ± 0.008 (HQC). This discrepancy can be further visualized by comparing the LC-MS/MS and ICP-MS equilibration time courses in Fig. 3, A and B. There are large fluctuations in K_RBC/PL_ throughout the time course and a concentration difference for the LC-MS/MS data; however, for the ICP-MS data, there is no concentration dependence, and K_RBC/PL_ remains consistently between 0.55 and 0.65. To investigate this further, we repeated the same experiment with sodium heparin-treated whole blood and saw that these results were in better agreement with the ICP-MS data. The comparison of K_2_EDTA treatment and sodium heparin treatment was evaluated for the entire library using pooled whole blood and an equilibration period of 2 hours based on the equilibrium results. The K_RBC/PL_ calculated are presented in Table 3 at both a high and low concentration for each anticoagulant. K_2_EDTA-treated whole blood shows a systemically lower partitioning into the RBC fraction across the library compared to sodium heparin.

**Table 3:** Effect of anticoagulant on RBC/PL partitioning equilibrium K_RBC/PL_ calculated by quantitative LC-MS/MS analysis (ICP-MS for Ga^3+^) for PL and RBC fractions in both sodium heparin and K_2_EDTA treated whole blood. Data are presented as average ± SEM, *n* = 3 per sample, per concentration

Library candidate	Heparin K_RBC/PL_		K_2_EDTA K_RBC/PL_	
	0.1 *μg/mL*	1 *μg/mL*	0.1 *μg/mL*	1 *μg/mL*
Ga^3+^	0.12 ± 0.01	0.11 ± 0.01	0.12 ± 0.01	0.11 ± 0.03
GaSal	0.61 ± 0.42	0.57 ± 0.19	0.22 ± 0.02	0.26 ± 0.03
GaSal3	1.08 ± 0.28	1.03 ± 0.24	0.28 ± 0.08	0.28 ± 0.04
GaSal4	3422 ± 435	8098 ± 1102	2288 ± 94	9220 ± 484
GaSal5	1.91 ± 0.57	1.34 ± 0.17	0.63 ± 0.36	0.52 ± 0.01
GaSal8	33.8 ± 5.7	37.4 ± 2.6	0.12 ± 0.09	0.10 ± 0.05
GaSal9	NC	NC	820 ± 59	13745 ± 3800
GaSal11	0.91 ± 0.19	1.15 ± 0.19	0.05 ± 0.03	0.05 ± 0.02
GaSal12	0.86 ± 0.31	1.48 ± 0.10	0.11 ± 0.06	0.13 ± 0.04
GaSal13	0.47 ± 0.22	0.59 ± 0.18	0.55 ± 0.35	0.08 ± 0.04
GaSal14	0.55 ± 0.07	1.86 ± 0.36	0.69 ± 0.10	1.65 ± 0.18
GaSal15	0.83 ± 0.01	1.24 ± 0.03	5.40 ± 0.13	3.81 ± 0.97

NC, not calculated due to unquantifiable signal in the plasma fraction.

To further show that this phenomenon was not due to a lot of effects from biological matrices, 6 selectivity lots (3 male and 3 female individual donors) of whole blood were analyzed for select analogs and showed good agreement between the ICP-MS and LC-MS/MS samples for sodium heparin samples (Table 4), but the K_2_EDTA treated lots repeat the same trend as the pooled lots assed in Table 3 (Table 5).

**Table 4:** Comparison of selectivity lots for selected library compounds K_RBC/PL_ calculated by quantitative LC-MS/MS analysis (ICP-MS for Ga^3+^) for PL and RBC fractions in sodium heparin-treated whole blood. Data are presented as average ± SEM, *n* = 6 biological replicates per compound

Compound	LC-MS/MS calculated K_RBC/PL_	ICP-MS calculated K_RBC/PL_
	1 *μg/mL*	1 *μg/mL*
Ga^3+^	ICP-MS Only	0.08 ± 0.01
GaSal	0.45 ± 0.03	0.89 ± 0.04
GaSal5	0.56 ± 0.03	0.36 ± 0.03
GaSal13	0.42 ± 0.03	0.48 ± 0.04
GaSal14	0.33 ± 0.04	0.34 ± 0.02

**Table 5:** Comparison of the effect of anticoagulant on RBC partitioning of selectivity lots of whole blood analyzed by LC-MS/MS K_RBC/PL_ calculated by quantitative LC-MS/MS analysis for PL and RBC fractions in both sodium heparin- and K_2_EDTA-treated whole blood. Heparin data are also presented in the previous table. Data are presented as average ± SEM, *n* = 6 biological replicates per compound. Significance was determined by 3-tailed Student’s *t* test

Compound	Heparin K_RBC/PL_	K_2_EDTA K_RBC/PL_	*P*-value
	1 *μg/mL*	1 *μg/mL*	
GaSal	0.45 ± 0.03	0.58 ± 0.02	.04
GaSal5	0.56 ± 0.03	0.16 ± 0.03	<.01
GaSal14	0.33 ± 0.04	0.08 ± 0.01	<.01

## 4 Discussion

### 4.1 Challenges in metallodrug development

Metallodrugs are a class of understudied compounds, and there is limited literature that describes the optimization and screening of metallodrug libraries. Although ICP-MS will provide the measurement of the metal core, these data will provide limited information on metabolite formation and potential release of the metal core from the scaffold. Moreover, LC-MS/MS is the gold standard analytical technique for large cohorts of bioanalytical studies due to specificity, sensitivity, and throughput. This article outlines differences in sample preparation and analytical methods important to be considered during metallodrug development.

### 4.2 First reported distribution of Ga^3+^ in RBC and PL

To our knowledge, this is the first study to detail the distribution of Ga^3+^ in PL and whole blood. Establishing b/p partitioning has emerged as a parameter to help model the volume of distribution for in vivo studies (Poulin et al, 2024). Gallium nitrate has shown efficacy in the treatment of *P*. *aeruginosa*; however, prolonged exposure results in renal toxicity (Hall et al, 1979; Krakoff et al, 1979; Choi et al, 2018). Additionally, free gallium will impact iron metabolism and transport, disrupting transferrin receptor-mediated endocytosis (Seligman et al, 1992; Chitambar, 2012). It is important to ensure the GaSal scaffold retains Ga^3+^ instead of releasing gallium to avoid similar toxicity issues. In turn, this would demonstrate the convincing superiority of GaSal compounds over previously clinically administered gallium nitrate (Ganite). Moreover, it is necessary to prove that preanalytical workflows do not artificially disrupt the intact scaffold.

### 4.3 Choice of anticoagulant is necessary for accurate quantitation

K_2_EDTA is a common anticoagulant used for sample collection in toxicity and PK studies. We have shown in Fig. 3, A and B that the calculated K_RBC/PL_ is lower when measured by LC-MS/MS instead of ICP-MS when K_2_EDTA is used as the anticoagulant. We hypothesize that this is due to the ability of EDTA to chelate metals. EDTA has been used therapeutically to chelate metals during toxic exposure (Glicklich et al, 2020; Layne et al, 2020). This is a closed system, so the mass balance of gallium should be consistent once equilibrium is reached for ICP-MS; however, using the LC-MS/MS data, we can observe that gallium detected as part of the salophen scaffold is inconsistent.

We see the distinct trend that the K_RBC/PL_ for K_2_EDTA samples exhibit a lower distribution in the RBC when treated with K_2_EDTA. The impact of anticoagulant on distribution of therapeutics has been demonstrated in other classes of drugs (Afolaranmi et al, 2010; Bayliss et al, 2019). Our data are consistent with K_2_EDTA chelating the compound, further bulking the scaffold, and increasing the molecular weight of the compound. This could result in decreasing passive diffusion or steric hindrance with key erythrocyte transporters. Quantitative results for K_RBC/PL_ were consistent between LC-MS/MS and ICP-MS for heparin-treated whole blood samples (Table 4). We have concluded that heparin is the preferred anticoagulant for sample collection during in vivo studies and this is supported by orthogonal metallodrug measurements by LC-MS/MS and ICP-MS.

### 4.4 Permeability experiments demonstrate a need for dosing considerations and further understanding of transporter activity

Based on the Caco-2 permeability assay, we have shown that the GaSal library tested would be a poorly bioavailable drug because all of the library compounds show lower P_app_ than minoxidil (Table 2, Supplemental Table 2). Although oral dosing is advantageous for patient access, it is a common clinical practice to deliver the antibiotic tobramycin via inhalation directly to the site of infection to improve therapeutic efficacy (Akkerman-Nijland et al, 2020; Marasini et al, 2022). This may be a promising approach for future dosing studies of GaSal analogs. Additionally, the large efflux for this class of compounds is very high, so it is possible that efflux transporter activity is dominating the drug movement, giving the appearance of poor permeability.

GaSal-2 and GaSal-7 were tested, but not able to be detected by LC-MS/MS for Caco-2 experiments. We hypothesized that the tertiary amine group is a promiscuous binding group, which causes nonspecific binding to other proteins present. Thus, in sample preparation, the compound is precipitated out of the supernatant still attached to the protein. This physiochemical property is also found in other compounds that share this structural moiety, such as imipramine and amitriptyline, which have been reported to be >90% PL protein bound (Pike and Skuterud, 1982; Zhang et al, 2012). Future compounds that contain this moiety should be analyzed by ICP-MS.

According to experimental permeability data provided herein, these compounds experimentally show little permeability from apical to basolateral. In the context of heme transport, this may be because key heme transporters are also responsible for the transport of the drug. Heme accumulation can be toxic to the cells, and it has been reported that heme transport can even occur against a concentration gradient to prevent cytotoxic effects in Caco-2 cell lines (Uc et al, 2004). Future studies should probe known heme transporters, solute carrier family 48 member 1 and MDR protein 5, because these are key proteins involved in heme trafficking (Reddi and Hamza, 2016). This is supported by the lack of impact of OCT1 and P-gp inhibitor cocktails on the P_app_ of GaSal. To our knowledge, although this study is the first to look at in vitro permeability assessment, in vivo permeability and bioavailability still need to be assessed in future experiments.

### 4.5 GaSal CYP inhibition may be a therapeutic benefit

Clinical isolates of *P*. *aeruginosa* have demonstrated regional variability for antibiotic susceptibility. Reports have also shown that the fluoroquinolone-based antibiotic ciprofloxacin has a large PK variation in patients with cystic fibrosis (Schultz et al, 2017; Akkerman-Nijland et al, 2020). Furthermore, there are recent studies that show CYP enzymes unique to *P*. *aeruginosa* that may impact cell signaling (Ueyama et al, 2005; Tooker et al, 2022). *Pseudomonas aeruginosa* is able to readily adapt to its environment, and it is hypothesized that these CYP enzymes help to metabolize antibiotic threats or dampen the host-response signaling molecules. Our microsomal data show that there is CYP inhibition when GaSal is incubated with 7-ethoxycoumarin. Although CYP inhibition was not considered in the initial design of the GaSal library, metabolic screening has helped to elucidate another potential mechanism of action for this drug program. Future experiments should examine if clearance of antibiotics is reduced in MDR clinical isolates in the presence of GaSal, to what extent inhibition occurs, and if this inhibition nonspecifically affects CYP enzymes or specific isoforms.

## 5 Conclusion

In this article, we detail the unique challenges of preclinical screening for metallodrug development. Understanding necessary preanalytical considerations is critical for accurate reporting. ICP-MS analysis alone has been used to characterize PK for clinical candidates; however, this assumes that the metallotherapeutic remains intact. By combining LC-MS/MS data sets with ICP-MS analysis, it is possible to thoroughly understand the characterization of the metallodrug profile with confidence because orthogonal analytical methods are employed. GaSal compounds show limited permeability; however, data indicate that further investigation is needed to characterize transporters involved in drug distribution. Blood/PL partitioning experiments indicate a difference in the distribution of Ga^3+^ and GaSal compounds, indicating that the scaffold does remain intact in a biorelevant matrix. Future studies will examine whether in vitro results are translatable to in vivo dosing, thus furthering the knowledge of in vitro/in vivo correlation of metal-based compounds. Ultimately, these approaches can be applied to other metallodrugs and aid in the future of metallotherapeutic drug development.

## Conflict of interest

F.X. and A.W. are co-founders of Galio Therapeutics, LLC.
