# Supplementary material for: Application of preclinical absorption, distribution, metabolism, elimination in vitro techniques for the characterization and compound library optimization of novel antibiotic gallium salophen
Source: Drug Metab Dispos. 2025 Apr 16;53(6):100080. doi: 10.1016/j.dmd.2025.100080 (PMC12264554; doi:10.1016/j.dmd.2025.100080)
Supplement: Supplementary Figures 1-4 and Supplementary Tables 1-4 [file mmc1.docx]

**Supplemental Information**

Application of pre-clinical ADME *in vitro* techniques for the characterization and compound library optimization of novel antibiotic gallium salophen (GaSal)

Samuel A. Krug, Aziza Frank, Lucia Hwang, Madison Worth, Kieran Johnson, Christine Rojas, Ludovic Muller, Sarah LJ Michel, Angela Wilks, Fengtian Xue, Maureen A. Kane*

University of Maryland Baltimore, School of Pharmacy

Department of Pharmaceutical Sciences

20 N Pine Street

Baltimore, MD 21201

**Supplemental Table 1**: List of LC-MS/MS transitions used in this study.

**Supplemental Figure 1:** Comparison of GaSal analyzed by DESI and ESI

**Supplemental Figure 2:** Representative MS1 Spectra for GaSal in source fragmentation

**Supplemental Figure 3:** Representative chromatographic shift for experimental logP determination using LC-MS/MS

**Supplemental Table 2**: Data summary of calculated Caco-2 permeability using known drug standards

**Supplemental Table 3:** Data summary of Caco-2 permeability for GaSal in the presence of inhibitor cocktails.

**Supplemental Figure 4:** Lucifer Yellow comparison with and without GaSal

**Supplemental Table 4:** B/P ratio in this study for Gallium compared to calculated B/P for other reported metals.

**Supplemental Table 1**: **List of LC-MS/MS transitions used in this study.** Control drugs are listed before GaSal Library compounds.

| **Compound** | ***m/z* transition** | **Cone**  **(V)** | **Collision**  **(eV)** |
| --- | --- | --- | --- |
| 7-ethoxycoumarin | 191.1 → 107.0 | 50 | 35 |
| Methotrexate | 455.1 → 307.9 | 50 | 20 |
| Minoxidil | 210.1 → 164.9 | 50 | 20 |
| Loperamide | 477.1 → 266.0 | 50 | 30 |
| Citalopram | 325.1 → 108.9 | 50 | 30 |
| Verapamil | 455.2 → 165.2 | 50 | 20 |
| GaSal | 383.3→ 69.0 | 60 | 30 |
| GaSal2 | 557.1 → 72.0 | 50 | 20 |
| GaSal3 | 641.1 → 114.0 | 60 | 50 |
| GaSal4 | 559.1 → 356.9 | 60 | 60 |
| GaSal5 | 619.2 → 414.8 | 60 | 40 |
| GaSal7 | 485.1 → 72.1 | 50 | 20 |
| GaSal8 | 460.1 → 114.1 | 25 | 20 |
| GaSal9 | 485.1 →383.8 | 80 | 25 |
| GaSal11 | 531.1 →414.8 | 60 | 60 |
| GaSal12 | 471.0 →295.0 | 50 | 35 |
| GaSal13 | 531.1 →414.8 | 60 | 60 |
| GaSal14 | 471.0 →295.0 | 50 | 35 |
| GaSal15 | 527.1 →414.7 | 50 | 35 |

**Supplemental Figure 1: Comparison of GaSal analyzed by DESI and ESI.** GaSal analyzed by DESI at a concentration of 1 µg/mL (TOP) vs infusion of GaSal at 1 µg/mL analyzed via ESI + mode. Gallium adducts are not seen in DESI analysis however, adducts can be seen via ESI + at 415/417, 435/437, 451/453, 461/463, 519/521 *m/z* in similar abundance to the expected precursor ion.

**Supplemental Figure 2:** **Example MS1 Spectra for GaSal in source fragmentation.**

Example MS1 Spectra for GaSal at cone voltage of 100 V and 150 V. Increase of 415/417 and 473/475 *m/z* abundance are consistent with scaffold breakdown while maintaining the characteristic gallium isotope peak. Additional adducts at 563/565, 583/585, 599/601, and 615/617 are consistent with previously reported metal salophen/salen compounds.

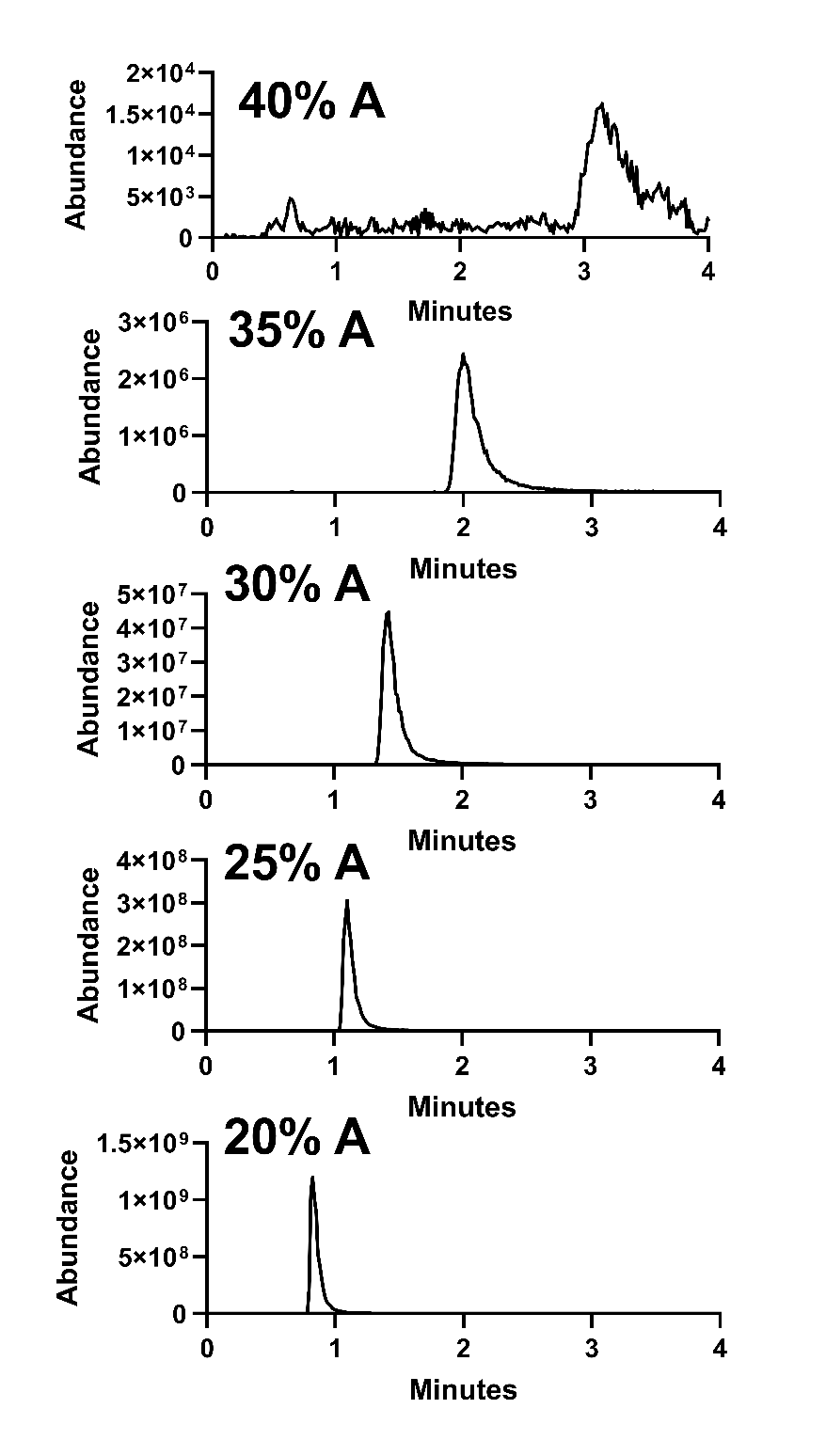
**Supplemental Figure 3:** **Representative chromatographic shift for experimental logP determination of GaSal3 using LC-MS/MS**. LC-MS/MS method was performed using isocratic elution with decreasing aqueous mobile phase composition. As seen in 40%A, the molecule is ionized very poorly and has a late retention time compared to the subsequent injections with a higher organic mobile phase composition.

**Supplemental Table 2**: **Data summary of calculated Caco-2 permeability using known drug standards.** Data summary of calculated Caco-2 permeability using known drug standard for low, moderate, and high permeability with the cell line used for library screening. Units of apparent permeability are x 10^-6^ cm/sec.

| **Analyte** | **Permeability Ranking** | **P_app_ Apical to Basolateral** | **P_app_ Basolateral to Apical** | **Efflux Ratio** |
| --- | --- | --- | --- | --- |
|  |  |  |  | B to A/ A to B |
| Methotrexate | Not Permeable | 0.02±0.001 | 0.03±0.01 | 1.46 |
| Minoxidil | Low | 1.12±0.04 | 3.29±0.54 | 2.92 |
| Loperamide | Moderate | 3.51±0.04 | 14.82±2.12 | 4.22 |
| Citalopram | High | 5.33±0.07 | 16±4.39 | 3 |

**Supplemental Table 3:** **Data summary of Caco-2 permeability for GaSal in the presence of inhibitor cocktails.** Units of apparent permeability are x 10^-6^ cm/sec.

| **Analyte** | **P_app_ Apical to Basolateral** | **P_app_ Basolateral to Apical** | **Efflux Ratio** |
| --- | --- | --- | --- |
|  |  |  | B to A/ A to B |
| GaSal | 1.20±0.01 | 13.6±1.78 | 11.3 |
| GaSal with PGP inhibitor cocktail | 1.33±0.03 | 16.79±3.86 | 12.6 |
| GaSal with OCT1 inhibitor cocktail | 1.81±0.03 | 14.73±3.26 | 8.1 |

**Supplemental Figure 4:** **Lucifer Yellow comparison with and without GaSal.** Representative data for the effect of metallodrug on Lucifer yellow emission. Measured fluorescence units with excitation at 485 nm and emission at 535 nm. Final concentration was 50 µM for lucifer yellow and 10 µM for GaSal. No significant difference was found when comparing the data using Student’s t-test.


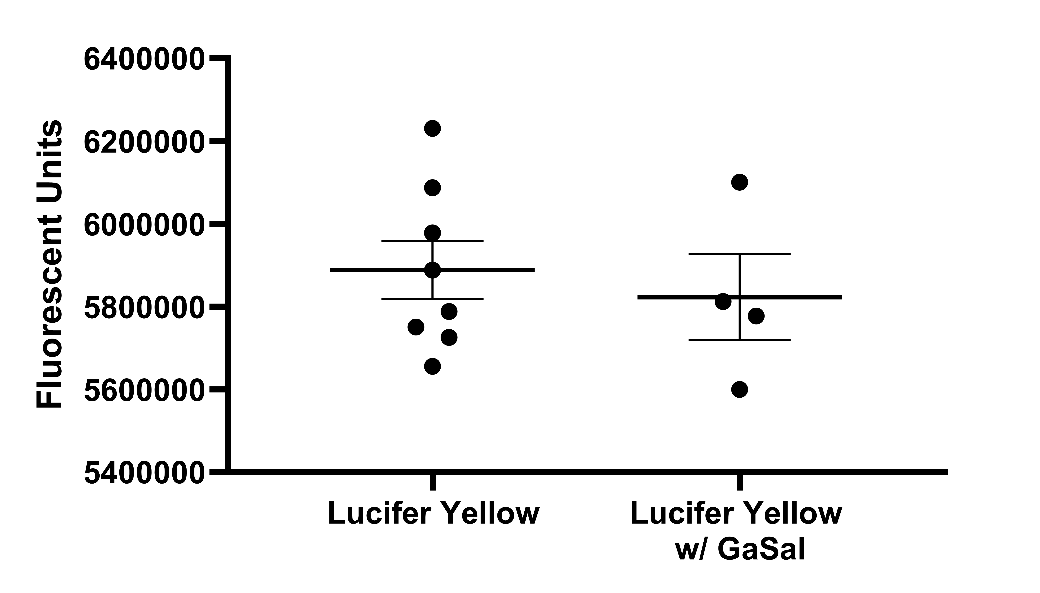


**Supplemental Table 4:** **B/P ratio in this study for Gallium compared to calculated B/P for other reported metals.** B/P is defined as the concentration of the metal in whole blood divided by the concentration of the metal in plasma.

| **Metal** | **K_RBC/PL_** | **Reference** |
| --- | --- | --- |
| Gallium | 0.3 | This study |
| Titanium | 0.7-1.0 | (Sarmiento-González et al., 2009) (Richardson et al., 2008) |
| Vanadium | 0.5-0.6 | (Heitland & Köster, 2006) (Heinemann et al., 2003) |
| Chromium | 1-3 | (Bonde & Christensen, 1991) |
| Manganese | 0.2 | (Smith et al., 2007) |
| Iron | 200-1200 | (Van den Bosch et al., 2001)) |
| Cobalt | 0.1; 1-2 | Wolterink (1955) (Lee & Wolterink, 1955) |
| Lead | 1.13 | (Cao et al., 2023) |
| Aluminum | 0.67 | (Cao et al., 2023) |
| Nickel | 0.50 | (Cao et al., 2023) |
| Copper | 0.82 | (Cao et al., 2023) |
| Zinc | 4.84 | (Cao et al., 2023) |
| Arsenic | 1.46 | (Cao et al., 2023) |
| Selenium | 1.34 | (Cao et al., 2023) |
| Strontium | 0.42 | (Cao et al., 2023) |
| Cadmium | 4.28 | (Cao et al., 2023) |

**Supplemental References**

Bonde, J. P., & Christensen, J. M. (1991). Chromium in biological samples from low-level exposed stainless steel and mild steel welders. *Archives of Environmental Health: An International Journal*, *46*(4), 225-229.

Cao, K., Zhang, J., Wang, G., Lin, X., Zhan, F., Wu, K.,…Liu, C. (2023). Associations of trace element levels in paired serum, whole blood, and tissue: an example of esophageal squamous cell carcinoma. *Environ Sci Pollut Res Int*, *30*(13), 38052-38062. <https://doi.org/10.1007/s11356-022-24960-z>

Heinemann, G., Fichtl, B., & Vogt, W. (2003). Pharmacokinetics of vanadium in humans after intravenous administration of a vanadium containing albumin solution. *British journal of clinical pharmacology*, *55*(3), 241-245.

Heitland, P., & Köster, H. D. (2006). Biomonitoring of 30 trace elements in urine of children and adults by ICP-MS. *Clinica Chimica Acta*, *365*(1-2), 310-318.

Lee, C.-C., & Wolterink, L. (1955). Urinary excretion, tubular reabsorption and biliary excretion of cobalt 60 in dogs. *American Journal of Physiology-Legacy Content*, *183*(1), 167-172.

Richardson, T. D., Pineda, S. J., Strenge, K. B., Van Fleet, T. A., MacGregor, M., Milbrandt, J. C.,…Freitag, P. (2008). Serum titanium levels after instrumented spinal arthrodesis. *Spine*, *33*(7), 792-796.

Sarmiento-González, A., Encinar, J. R., Marchante-Gayón, J. M., & Sanz-Medel, A. (2009). Titanium levels in the organs and blood of rats with a titanium implant, in the absence of wear, as determined by double-focusing ICP-MS. *Analytical and Bioanalytical Chemistry*, *393*, 335-343.

Smith, D., Gwiazda, R., Bowler, R., Roels, H., Park, R., Taicher, C., & Lucchini, R. (2007). Biomarkers of Mn exposure in humans. *Am J Ind Med*, *50*(11), 801-811. <https://doi.org/10.1002/ajim.20506>

Van den Bosch, G., Van den Bossche, J., Wagner, C., De Schouwer, P., Van De Vyvere, M., & Neels, H. (2001). Determination of iron metabolism-related reference values in a healthy adult population. *Clinical chemistry*, *47*(8), 1465-1467.
